# Molecular and Microbiological Insights on the Enrichment Procedures for the Isolation of Petroleum Degrading Bacteria and Fungi

**DOI:** 10.3389/fmicb.2018.02543

**Published:** 2018-10-30

**Authors:** Giulia Spini, Federica Spina, Anna Poli, Anne-Laure Blieux, Tiffanie Regnier, Carla Gramellini, Giovanna C. Varese, Edoardo Puglisi

**Affiliations:** ^1^Department for Sustainable Food Processes, Università Cattolica del Sacro Cuore, Piacenza, Italy; ^2^Department of Life Sciences and Systems Biology, Mycotheca Universitatis Taurinensis, University of Turin, Turin, Italy; ^3^Satt Grand-EST, Maison Règionale de l’Innovation, Dijon, France; ^4^ARPAE-Emilia Romagna, Bologna, Italy

**Keywords:** bioremediation, crude oil, soil contamination, enrichment culture, metagenomics, bacteria, fungi

## Abstract

Autochthonous bioaugmentation, by exploiting the indigenous microorganisms of the contaminated environment to be treated, can represent a successful bioremediation strategy. In this perspective, we have assessed by molecular methods the evolution of bacterial and fungal communities during the selective enrichment on different pollutants of a soil strongly polluted by mixtures of aliphatic and polycyclic hydrocarbons. Three consecutive enrichments were carried out on soil samples from different soil depths (0–1, 1–2, 2–3 m), and analyzed at each step by means of high-throughput sequencing of bacterial and fungal amplicons biomarkers. At the end of the enrichments, bacterial and fungal contaminants degrading strains were isolated and identified in order to (i) compare the composition of enriched communities by culture-dependent and culture-independent molecular methods and to (ii) obtain a collection of hydrocarbon degrading microorganisms potentially exploitable for soil bioremediation. Molecular results highlighted that for both bacteria and fungi the pollutant had a partial shaping effect on the enriched communities, with paraffin creating distinct enriched bacterial community from oil, and polycyclic aromatic hydrocarbons generally overlapping; interestingly neither the soil depth or the enrichment step had significant effects on the composition of the final enriched communities. Molecular analyses well-agreed with culture-dependent analyses in terms of most abundant microbial genera. A total of 95 bacterial and 94 fungal strains were isolated after selective enrichment procedure on different pollutants. On the whole, isolated bacteria where manly ascribed to *Pseudomonas* genus followed by *Sphingobacterium*, *Bacillus*, *Stenothrophomonas*, *Achromobacter*, and *Serratia*. As for fungi, *Fusarium* was the most abundant genus followed by *Trichoderma* and *Aspergillus*. The species comprising more isolates, such as *Pseudomonas putida*, *Achromobacter xylosoxidans* and *Ochromobactrum anthropi* for bacteria, *Fusarium oxysporum* and *Fusarium solani* for fungi, were also the dominant OTUs assessed in Illumina.

## Introduction

Oil hydrocarbons are the most widespread environmental pollutants, including n-alkanes, cycloalkanes, and polycyclic aromatic hydrocarbons (PAHs) that have been regarded as serious ecological and public health concerns (Bao et al., 2012). Oil contamination of ecosystems is a serious issue associated with crude oil drilling, transportation, refining, and related activities which demands immediate attention for restoration. Crude petroleum oil is a complex mixture of hydrocarbons mainly composed of saturated and aromatic hydrocarbons, asphaltenes and resins (Moubasher et al., 2015). Aliphatic hydrocarbons consist of readily biodegradable n-alkanes followed by less biodegradable branched and cyclic alkanes (Das and Chandran, 2011). Similarly, PAHs are compounds with two or more aromatic rings that are the most recalcitrant components present at high percentages in crude oil (Haritash and Kaushik, 2009).

Due to the public awareness and to the strict legal constraints on the release of pollutants into the environment, it is necessary to find effective and affordable technologies for the treatment of oil industrial wastes. Bioremediation is a biological approach that relies on the metabolic potential of microorganisms to remove contaminants (Maiti et al., 2008; Megharaj et al., 2011; Hara et al., 2013). The use of bioremediation techniques proved economical, environmentally friendly and flexible (Obi et al., 2016). In this prospect, bioremediation is gaining more and more importance as constructive approach for the remediation of polluted sites. Several studies reported the catabolic abilities of indigenous microorganisms such as fungi, bacteria and algae to degrade hydrocarbons (Dean-Ross et al., 2002; Bundy et al., 2004; Maiti et al., 2008; Wang et al., 2011; Badr El-Din et al., 2014). These microorganisms, adapted to the contaminated environments, are equipped with specific enzyme systems that enable them to use hydrocarbons as sole carbon source. Different hydrocarbon degrading microorganisms such as bacteria and archaea have been found in hydrocarbons contaminated environments (Alonso-Gutierrez et al., 2009; An et al., 2013; Hazen et al., 2013; Head et al., 2014; Fowler et al., 2016). The structure of the microbial community in a soil, influences deeply the degree of oil hydrocarbons degradation. Liu et al. (2011) observed that, at the early stage of remediation, the bacterial community was responsible for the degradation of the saturated and partially aromatic hydrocarbons; the fungal community instead became dominant in decomposing the polar hydrocarbons fraction in post-remediation. Generally, thanks to the variety of extracellular enzymes and fungal hyphae, fungi are the first key players in degrading available contaminants and recalcitrant polymers (Wick et al., 2007; Fernandez-Luqueno et al., 2010; Deshmukh et al., 2016). Fungal mobilization and degradation of contaminants contribute to release bioavailable intermediates on which, in a later stage, the bacterial community can act more easily (Smits et al., 2005; Scullion, 2006; Leonardi et al., 2008; Yuan et al., 2018). Therefore, in order to characterize and to monitor the native microbial community, the dynamics and the functional potential of both bacteria and fungi in polluted ecosystems is essential for the development of a successful bioremediation strategy (Atlas, 1981; Head et al., 2014; Covino et al., 2016).

The success of on-site bioremediation, employing native microbial populations, could be voided by imbalanced nutrients and/or adverse factors (temperature, moisture content, pH, availability of electron donor and/or acceptor, high pollutant concentration, etc.), which are common in contaminated sites (Smith et al., 2015). Bioremediation can be accomplished by either boosting the growth of the indigenous microbial community through biostimulation or by introducing naturally occurring microorganisms with excellent catabolic abilities (bioagumentation) that are adapted to the ecological conditions of the site (Agnello et al., 2016).

Many investigators have reported successful bioremediation, where biostimulation with the addition of appropriate nutrients (N and/or P), to avoid metabolic limitations, resulted in an improved metabolic activity of indigenous microorganisms (Yu et al., 2005; Ghaly and Yusran, 2013; Suja et al., 2014; Smith et al., 2015). On the other hand, autochthonous bioagumentation, based on the re-inoculation in polluted sites of indigenous microorganisms previously enriched under laboratory conditions, enhanced the microbial activities, thus improving the degradation of hydrocarbons (Dueholm et al., 2015). In order to provide an inoculum for bioaugmentation, the isolation of microorganisms in pure culture from these contaminated environments is fundamental. However, due to the complexity of crude oil, a microbial consortium composed of microorganisms endowed with diverse metabolic capacities and syntrophic interactions would work better than a pure culture. Several reports demonstrated the better metabolic versatility of mixed cultures in using hydrocarbon pollutants as sole carbon source in comparison to pure cultures (Cerqueira et al., 2011; Das and Chandran, 2011). In laboratory conditions, bacterial, and fungal co-culture(s) showed improved degradation rates of diesel oil and of polyacyclic romatic hydrocarbons (PAHs) (Wang et al., 2011). Hence, catabolic interactions among different microbial groups during biodegradation is extremely important (Atlas, 1981; Varjani et al., 2015). Although identifications and characterizations of the microorganisms involved in the degrading processes are available (Desai and Banat, 1997; Rojo, 2009), less is known on the biodiversity and dynamics of the native hydrocarbons-degrading microbial community of a contaminated soil, especially during the enrichment process applied to isolate the most effective strains (Omrani et al., 2018). The development of effective bioremediation strategies requires an extensive understanding of the resident microorganisms of these habitats. Recent techniques such as high-throughput sequencing (HTS) have greatly facilitated the advancement of microbial ecological studies in oil-polluted sites.

In the present work we have assessed by molecular methods the evolution of bacterial and fungal communities during the enrichment on different pollutants of a soil strongly polluted by mixtures of aliphatic and polycyclic hydrocarbons. Three consecutive enrichments were carried out on soil samples from different soil depths (0–1, 1–2, 2–3 m), and analyzed at each step by means of HTS of bacterial and fungal amplicons biomarkers. At the end of the enrichment, bacterial, and fungal contaminants degrading strains were cultivated and identified.

Main aims of the work were: (i) to assess the effect of different pollutants used as sole carbon during a selective enrichment procedure source on the diversity of the microbial community of crude oil contaminated soil (ii) to compare the composition of enriched communities by culture-dependent and culture-independent molecular methods and (iii) to obtain a collection of hydrocarbon degrading fungi and bacteria potentially exploitable for soil bioremediation.

## Materials and Methods

### Sampling and Soil Characteristic

Crude oil contaminated soil samples from an area of the industrial SIN (Site of National Interest) located in Fidenza (Emilia-Romagna, Italy) were collected at three different depths (0–1, 1–2, and 2–3 m). The polluted site (about 80,000 m^2^ wide) has a long history of industrial exploitation and it is contaminated by a variety of pollutants such as Benzene-Toluene-Ethylbenzene-Xylene (BTEX), n-alkanes and PAHs.

The chemical composition of the soil samples is described in Supplementary Table S1.

### Target Organic Contaminants

Target organic contaminants were chosen based on literature data and on the chemical characterization of the contaminated area of Fidenza SIN. All chemicals were purchased by Sigma-Aldrich (Germany). Benzene was selected as representative of BTEX. Pyrene, phenanthrene, and naphthalene were selected as representatives of 4, 3, and 2 rings PAHs. Stock solutions were prepared in methanol for naphthalene (20 mg/mL) and in ethanol 95% for phenanthrene (15 mg/mL) and for pyrene (5 mg/mL). Paraffin oil, representative of alkanes, and crude oil mixture from the contaminated Fidenza site were used at a final concentration of 1% v/v.

### Microcosm Enrichments and Microbial Isolation

Different enrichment cultures were set up using the polluted soil as inoculum and the above-mentioned crude oil hydrocarbons components as the sole carbon source. One hundred g of each soil sample at the three depths (namely, S1, S2, and S3) were added to 900 mL sterile Mineral Medium (MM) supplemented with the target analyte as sole carbon source. To provide the proper microelement amount for bacterial and fungal growth, two mineral media were used: M9 mineral medium (Difco, Sparks, MD, United States) for bacteria and Czapek for fungi.

The flasks were incubated on a rotary shaker at 30°C and 180 rpm for bacteria and at 24°C and 120 rpm for fungi. After 7 days, 5 mL of the culture was transferred to another flask with MM and the corresponding target analyte: benzene (50 ppm); naphthalene (200 ppm), phenanthrene (200 ppm), pyrene (200 ppm); paraffin oil (1% v/v), and crude oil (1% v/v). Three consecutive subcultures were performed in the same conditions.

Each fungal and bacterial enrichment culture was plate on solid MM containing each pollutant as sole carbon source. To isolate pure microbial cultures, morphologically different colonies were selected and transferred to Malt Extract Agar (MEA) plates for fungi and Tryptone Soy Agar (TSA) plates for bacteria.

### Molecular Characterization of Fungal and Bacterial Isolates

#### Bacteria

DNA from isolate purified colonies was extracted using Microlysis kit (Labogen, London, United Kingdom) according to the manufacturer’s protocol. The isolates were screened by randomly amplified polymorphic DNA-polymerase chain reaction (RAPD-PCR) using the single stranded oligonucleotides primer RAPD2 (5′-AGCAGCGTGG-3′) (Rebecchi et al., 2015) and GTG-5 (5′-GTG GTG GTG GTG GTG-3′) (Versalovic et al., 1994). The PCR fragment profiles were digitally captured using the BioImaging System Gene Genius and pattern analysis was performed with the Fingerprinting II software (Bio-Rad Laboratories, Hercules, CA, United States). The similarity in the profiles of bands was based on the Pearson correlation coefficient and the cluster analyses were performed by unweighted pair group method with arithmetic mean (UPGMA). A correlation coefficient of 70% was arbitrarily selected to distinguish the clusters, and one representative for each cluster was amplified using the primers P0 (5′-GAG AGT TTG ATC CTG GCT-3′) and P6 (5′-CTA CGG CTA CCT TGT TAC-3′) (Di Cello and Fani, 1996). PCR products were visualized by electrophoresis on 2.5% agarose gel in Tris-Acetate-EDTA (TAE). The PCR amplicons of approximately 1.5 kb, corresponding to the size of the full 16S rRNA gene were purified using the NucleoSpin gel and PCR clean-up according to the package insert (Macherey-Nagel, DE) and sequenced at the GATC Biotech (Germany). The taxonomical identification of sequences was performed using BLAST (Basic Local Alignment Search Tool) (Altschul et al., 1997) and by alignment against the Ribosomal Database Project (RDP) database using the Naïve Bayesian Classifier (Wang et al., 2007).

#### Fungi

Genomic DNA of each strains was extracted from about 100 mg of mycelium scraped from the MEA petri dishes using the NucleoSpin^®^ Plant II kit (Macherey-Nagel), according to the manufacturer’s instruction. The quality and quantity of extracted DNA was measured spectrophotometrically by Infinite M200 (TECAN Trading, Austria). De-replication of fungal isolates was performed by using the minisatellite core sequence derived from the wildtype phage M13 (5′-GAG GGT GGC GGT TCT-3′) as specific primer to amplify variable number tandem repeat (VNTR) (Poli et al., 2016). Molecular identification of each fungal isolate was carried out by amplification of specific markers (White et al., 1990; Glass and Donaldson, 1995; Bensch et al., 2012). PCR products were visualized by electrophoresis on a 1.5% agarose gel stained with ethidium bromide in Tris-Borate-EDTA (TBE). PCR products were purified and sequenced at Macrogen Europe (Amsterdam, Netherlands). Consensus sequences were obtained by using Sequencer 5.0 (Gene Code Corporation). Taxonomic assignments were inferred by querying with the Blastn algorithm (default setting), hosted at NCBI (National Center for Biotechnology Information), the newly generated sequences against the nucleotide database of NCBI (GenBank). Pairwise alignments were also performed against the CBS-Knaw Fungal Biodiversity Centre (Centraalbureau voor Schimmel cultures) database. Similarity values equal or higher than 98% (e-value > e-100) were considered reliable; results were confirmed morphologically.

### DNA Extraction From Original Soils and Enrichments

Total microbial DNA was extracted from original soil samples and from each enrichment step of fungal and bacterial culture/microcosms with the PowerLyzer PowerSoil kit (MoBIO Laboratories, Inc., Carlsbad, CA, United States) according to the manufacturer’s instructions. DNA purity was checked with electrophoresis on a 0.8% agarose gel, while quantification was performed with the Quant-iTTM HS ds-DNA assay kit (Invitrogen, Paisley, United Kingdom) method in combination with the QuBit^TM^ fluorometer.

### Molecular Analyses of Microbial Diversity

#### Illumina Sequencing of 16S PCR Amplicons

For bacteria, PCR amplicons covering the V3–V4 regions of the 16S rRNA were analyzed in Illumina MiSeq with V3 chemistry in 300 bp paired-reads mode. PCR reactions were performed using indexed primer pairs 343F (5′-TACGGRAGGCAGCAG-3′) and 802R (5′-TACNVGGGTWTCTAATCC-3′), as described in Vasileiadis et al. (2015). A multiplexing strategy was employed to analyze several amplicon samples simultaneously in the same sequencing run. A nine nucleic acids extension was added to the 5′ end of the forward primer, where the first seven bases served as a tag to identify to each sample, and the following two bases were a linker designed not to match bacterial sequences in the same position according to RDP entries. In order to reduce possible biases related to the primer extension, the two step-PCR approach described in Berry et al. (2011) was adopted. The PCR conditions were set as described by Vasileiadis et al. (2015). The final PCR products were checked on 0.8% agarose gel and pooled in equimolar amounts according to QuBit measurements. The final pool was cleaned with the SPRI (Solid Phase Reverse Immobilization Method) using the Agencourt^®^ AMPure^®^ XP kit (Beckman Coulter, Milan, Italy). Finally, the pool was sequenced by BioFab Company (Rome, Italy) with a MiSeq Illumina instrument (Illumina, Inc., San Diego, CA, United States) operating with V3 chemistry and producing 300 bp paired-reads.

#### Illumina Sequencing of ITS PCR Amplicons

The nr ITS2 region was amplified from all DNA by means of a semi-nested PCR approach. In the first PCR, the nr ITS (ITS1-5.8S-ITS2) was amplified with universal primers ITS1F-ITS4 (White et al., 1990). For the second PCR, ITS3 and ITS4 (White et al., 1990) tagged primers were used to amplify the ITS2 region of each DNA sample (Voyron et al., 2017).

PCR products were pooled and purified using Wizard SV Gel and PCR Clean-Up System (Promega) following the manufacturer’s instructions. After quantification with Qubit 2.0 (Thermo Fisher Scientific, Waltham, MA, United States), the purified PCR products were mixed in equimolar amounts to prepare sequencing libraries. The libraries were paired-end sequenced using the Illumina MiSeq technology (2 bp × 250 bp) by IGA Technology Services S.r.l. Unipersonale (Udine, Italy).

### Sequences Data Preparation, Bioinformatics, and Statistical Analyses

The first steps for sequences processing and filtering were the same for both 16S and ITS amplicons. Raw paired Illumina sequences were merged with the “pandaseq” script (Masella et al., 2012) with a minimum overlap of 30 bp between read pairs and 2 maximum allowed mismatches. Sequences were multiplexed according to sample indexes and primers with the fastx-toolkit^1^. Both bacterial and fungal amplicons were analyzed with taxonomy-based and OTU-based analyses: in the first case, all sequences were individually classified at taxonomical level against relevant database (GreenGenes for bacteria, UNITE for fungi), while in the OTU-based analyses, sequences were grouped at 97% similarities. For 16S amplicons, both operational taxonomic units (OTUs) and taxonomy-based matrixes were produced with a pipeline in Mothur (Schloss et al., 2009). For ITS amplicons, taxonomy-based analyses were also performed in Mothur, whereas OTUs were determined in UPARSE (Edgar, 2013). The reason for this discrepancy was that no aligned databases are available for ITS, and the OTU-clustering method implemented in Mothur does not allow analysis of sequences that have dissimilar lengths, which was the case with the ITS amplicons.

For bacterial sequences, Mothur v.1.39.5 (Schloss et al., 2009) was applied in order to remove sequences with large homopolymers (≥10), sequences that did not align within the targeted V3–V4 region, chimeric sequences (Edgar et al., 2011) and sequences that were not classified as bacterial after alignment against the Mothur version of the RDP training data set. The resulting high- quality sequences were analyzed with Mothur and R^2^ following the OTU and the taxonomy-based approach. Sequences were first aligned against the SILVA reference aligned database for bacteria (Pruesse et al., 2007) using the NAST algorithm and a kmer approach (Schloss, 2010) and then clustered at the 3% distance using the average linkage algorithm. OTUs were classified into taxa by alignment against the Greengenes database (McDonald et al., 2012).

ITS taxonomy-based analyses were conducted in Mothur: sequences shorter than 120 bp were discarded. We discarded homopolymers > 10 bp and chimeras, which were identified with the UCHIME algorithm implemented in Mothur, with the UNITE database version 6 as reference. The same database was used to classify the retained sequences and to eliminate non-fungal sequences. OTUs were produced in USEARCH with the –fastx_uniques and - cluster_otus commands. Sequences that did not belong to fungi were identified with the sintax command against the Utax reference database and discarded.

The OTU- and taxonomy-based matrixes obtained were analyzed in R to estimate the associated α and β diversity of the samples. The Good’s coverage estimate was calculated to assess the “percentage diversity” captured by sequencing. The most abundant OTUs identified were confirmed with BLAST (Basic Local Alignment Search) searches against the GenBank and the RDP database.

## Results

### Hydrocarbon Degrading Bacterial and Fungal Strains

A collection of hydrocarbon-degrading bacterial and fungal strains was isolated from microcosms enriched on each pollutant (benzene, paraffin, pyrene, naphthalene, phenanthrene, and crude oil) and from three depths of the contaminated soil of the SIN in Fidenza. Molecular de-replication of isolated colonies resulted in 95 and 94 unique colonies for bacteria and fungi, respectively. Molecular identification of the strains was inferred by 16S rDNA and ITS sequencing and is reported in Table 1.

**Table 1:** Identification of bacteria and fungi isolated from benzene, paraffin, crude oil, naphthalene, pyrene, and phenanthrene enrichment microcosms.

	Bacterial isolates	Fungal isolates
Benzene	*Acinetobacter calcoaceticus (1)*	*Acremonium sclerotigenum (1)*
	*Bacillus subtilis (1)*	*Aspergillus creber (1)*
	*Benzo[a]pyrene-degrading bacterium (1)*	*Bjerkabndera adusta (1)*
	*Cellulosimicrobium* sp.*(1)*	*Eutypella scoparia (1)*
	*Pseudomonas mosselii (2)*	*Fusarium oxysporum (1)*
	*Pseudomonas putida (1)*	*Fusarium solani (5)*
	*Pseudoxanthomonas indica (1)*	*Irpex lacteus (1)*
	*Rhizobium petrolearium (1)*	*Pseudoallescheria boydii (2)*
	*Sphingobacterium* sp. (*3)*	*Scedosporium apiospermum (1)*
	*Stenotrophomonas acidaminiphila (2)*	*Scedosporium dehoogii (1)*
	*Stenotrophomonas maltophilia (1)*	
Paraffin	*Acholeplasma vituli (1)*	*Clonostachys rosea (1)*
	*Achromobacter xylosoxidans (3)*	*Fusarium oxysporum (5)*
	*Bacillus subtilis (3)*	*Fusarium solani (2)*
	*Bacillus xiamenentis (1)*	
	*Cupriavidus campinensis (1)*	
	*Gordonia rubripertincus (1)*	
	*Helicobacter* sp. *(1)*	
	*Paenibacillus* spp. (*1)*	
	*Pseudomonas putida (7)*	
	*Pseudoxanthomonas mexicana (3)*	
	*Sphingobacterium multivorum (6)*	
	*Stenotrophomonas acidaminiphila (1)*	
Crude oil	*Pseudomonas aeruginosa (1)*	*Aspergillus jensenii (1)*
	*Pseudomonas fluorescens (2)*	*Aspergillus protuberus (1)*
	*Serratia marcescens (3)*	*Aspergillus versicolor (6)*
		*Cladosporium cladosporioides (1)*
		*Cladosporium perangustum (2)*
		*Epicoccum nigrum (1)*
		*Penicillium crustosum (1)*
		*Trichoderma harzianum (1)*
		*Wallemia mellicola (1)*
Naphtalene	*Achromobacter* sp. *(1)*	*Aspergillus sclerotiorum (1)*
	*Pseudomonas fluorescens (4)*	*Aspergillus sydowii (1)*
	*Pseudomonas putida (2)*	*Aureobasidium pullulans (1)*
	*Pseudomonas* sp. *(7)*	*Cladosporium cladosporioides (1)*
	*Pseudomonas veronii (3)*	*Eutypella scoparia (1)*
	*Sphingobacterium multivorum (1)*	*Fusarium oxysporum (1)*
	*Stenotrophomonas maltophilia (1)*	*Penicillium brevicompactum (1)*
		*Penicillium catenatum (1)*
		*Scedosporium apiospermum (1)*
		*Sulcatispora acerina (1)*
		*Trametes gibbosa (1)*
Pyrene	*Ochrobactrum anthropi (1)*	*Aspergillus waksmanii (1)*
	*Pseudomonas fluorescens (4)*	*Cladosporium cladosporioides (2)*
	*Pseudomonas putida (1)*	*Fusarium oxysporum (2)*
	*Pseudomonas* sp. *(4)*	*Fusarium solani (9)*
		*Fusarium solani/keratoplasticum (3)*
		*Hypocrea lixii (2)*
		*Polyporus gayanus (1)*
		*Trichoderma harzianum (3)*
Phenanthrene	*Achromobacter* sp. *(1)*	*Epicoccum nigrum (1)*
	*Pseudomonas fluorescens (3)*	*Fusarium oxysporum (6)*
	*Pseudomonas putida (6)*	*Fusarium solani (6)*
	*Pseudomonas* sp. *(5)*	*Fusarium solani/keratoplasticum (2)*
		*Hypocrea lixii (2)*
		*Trichoderma harzianum (5)*


*The number of isolates per species is indicated in parenthesis.*

For bacteria, sequence similarity searches in NCBI GenBank and RDP database revealed the presence of cultivable members of different genera under the phyla Proteobacteria, Firmicutes, and Sphingobacteria. On the whole, the bacteria isolated where ascribed mainly to 12 genera belonging to both Gram-negative and Gram-positive. *Pseudomonas* was the most abundant genera (56.8%) followed by *Sphingobacterium* (12%), *Bacillus* (6%), *Stenothrophomonas* (6%), *Achromobacter* (6%), and *Serratia* (3%). Most of the bacterial strains (46) were isolated from enrichments inoculated with soil S2 (Supplementary Table S2): the most represented bacterial genera in soil S2 were *Pseudomonas* (23 isolates), *Serratia* (3 isolates), and *Sphingobacterium* (9 isolates). A lower number of bacterial strains was isolated from enrichments of soil S1 and S3 (Supplementary Table S2). A total of 26 isolates were obtained from soil S1 and were mainly affiliated to the genera *Bacillus*, *Pseudomonas* and *Pseudoxanthomonas*, while the 23 bacterial strains isolated from the deepest soil S3 were mainly affiliated to the genus *Pseudomonas* (Supplementary Table S2). The influence of pollutants used in the enrichments on the distribution of bacterial species is reported in Table 1. The highest number of bacterial isolates was obtained from paraffin (29 isolates), followed by naphthalene (19 isolates), benzene (15 isolates), and phenanthrene (15 isolates) microcosms; 10 strains were isolated in the presence of pyrene and only six strains were isolated in the presence of crude oil (Table 1). The genus *Pseudomonas* dominated all the microcosms enriched on pollutants. Benzene and paraffin enrichments showed the highest diversity of bacterial genera. In particular, benzene enrichment selected also bacterial species of the genera *Sphingobacterium* and *Stenotrophomonas*, while paraffin enrichment resulted also in the isolation of species of *Bacillus*, *Achromobacter*, and *Pseudoxanthomonas*. Beside *Pseudomonas*, *Serratia*, and *Ochromobactrum* were the most abundant genera in crude oil and pyrene microcosms (Table 1). At species level, *Pseudomonas putida* was dominant and was isolated in the presence of all the tested pollutants. Other frequently isolated species were *Pseudomonas fluorescence* (four pollutants), *Stenotrophomonas maltophilia (*two pollutants), *Stenotrophomonas acidaminiphila* (two pollutants), *Bacillus subtilis* (two pollutants), and *Sphingobacterium multivorum* (two pollutants). Three strains of *Serratia marcescens* were isolated only from the enriched microcosms with the crude oil of the contaminated site (Table 1).

Fungal species with their relative abundance are reported in Supplementary Table S3, according to the soil depth and in Table 1 according to the pollutant used in the enrichments. Isolated fungi belonged mostly to the phylum Ascomycota, and only 5% to Basidiomycota; Mucoromycota were not isolated. Most of the strains were isolated from enrichments inoculated with contaminated soils S1 (35 strains) and S3 (36 strains); a lower number of isolates (23) were retrieved from the contaminated soil S2 (Supplementary Table S3). In general, *Fusarium* was the most abundant genus (43.8%) followed by *Trichoderma* (and its teleomorph *Hypocrea lixii*) (13.8%) and *Aspergillus* (11.0%). The majority of the isolates detected in soils S1 and S3 were indeed ascribable to these three genera, while in S2 only 44.4% of the strains were affiliated to the genus *Fusarium* and 16.7% belonged to the genus *Cladosporium* (Supplementary Table S3).

The retrieval of fungal species was influenced by the pollutant used as sole carbon source in the enrichments, as reported in Table 1. The highest number of isolates was obtained from pyrene and phenanthrene (23-22 isolates) followed by benzene and crude oil (15 isolates); 11 strains were isolated in the presence of naphthalene and only eight strains were isolated in the presence of paraffin oil (Table 1). With the only exception of crude oil, the genus *Fusarium* was the most abundant in each pollutant. Benzene and paraffin enrichments revealed the highest variety of genus and species.

### Molecular Analyses of Bacterial and Fungal Communities

Illumina HTS of PCR amplicons of the 16S V3–V4 region resulted in 1,991,866 sequences, which were reduced to 1,742,447 after the exclusion of homopolymers, of sequences <380 bp, sequences not aligning to the targeted V3–V4 regions, chimera and sequences not classified as bacterial after alignment against the RDP 16S training set. In order to reduce biases in diversity estimates related to the analyses of samples with different number of sequences, rarefaction to a common minimum number of 10,065 sequences per sample was carried out (Gihring et al., 2012). The rarefaction step resulted in the loss of only 5 out of 82 samples, while retaining a sequencing depth able to depict most of the bacterial diversity in the samples, as indicated by an average Good’s coverage of 90.4%.

As for fungi, Illumina sequencing of ITS2 amplicons resulted in 795,742 high quality sequences. Taxonomy-based analyses were performed in Mothur and consisted in a screening to remove homopolymers, sequences shorter than 120 bp, chimera, and sequences that were not correctly classified as fungal after alignment against the UNITE v6 database. Rarefaction was performed with a common number of 5,001 sequences per sample. The coverage of fungal diversity was not affected by this rarefaction step: average Good’s coverage was 99.7%, thus showing that the sequencing effort covered the totality of fungal biodiversity in the samples. OTU-based analyses for fungi were performed in USEARCH and resulted in a very similar number of sequences per sample (4,942) and a total of 188 fungal OTUs.

**FIGURE 1:**
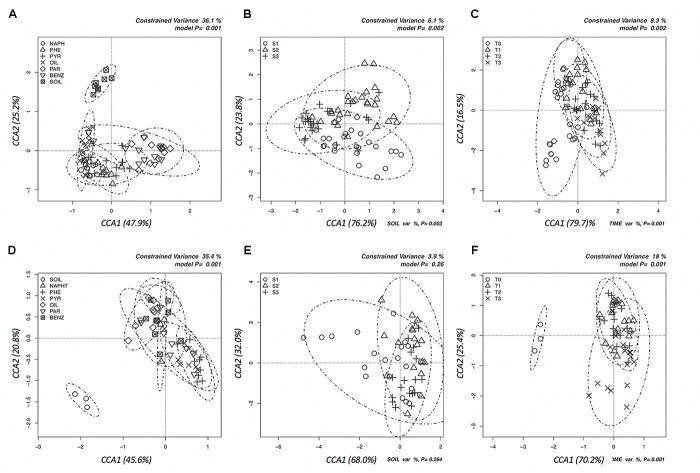
Canonical correspondence analyses (CCAs) to test the significance of the effects of pollutant **(A,D)**, soil depth **(B,E),** and time **(C,F)** on the total structure of bacterial and fungal communities as determined by the relative abundances of all the OTUs resulting by Illumina sequencing of bacterial 16S and fungal ITS amplicons. **(A–C)** Graphs in the first line are for bacteria, **(D–F)** are for fungi.

**FIGURE 2:**
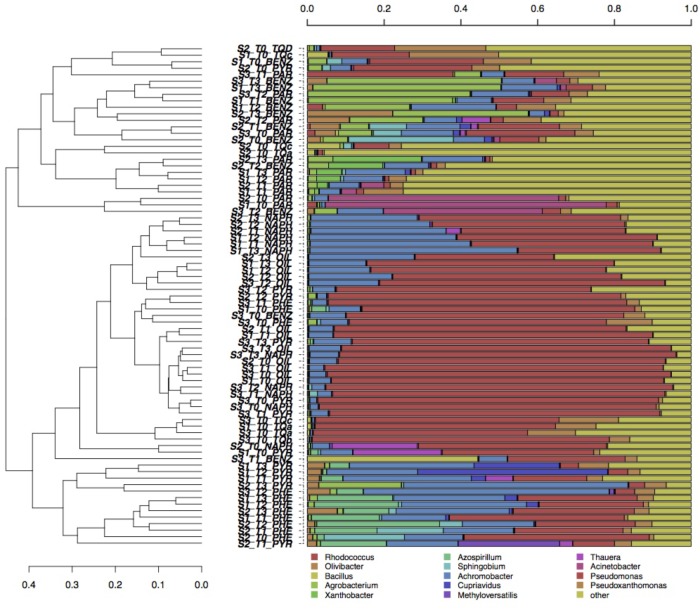
Hierarchical clustering of bacterial 16S sequences classified at the genus level. Only taxa participating with >20% in at least one sample are shown, while taxa with lower participation are grouped in the “other” sequence group.

**FIGURE 3:**
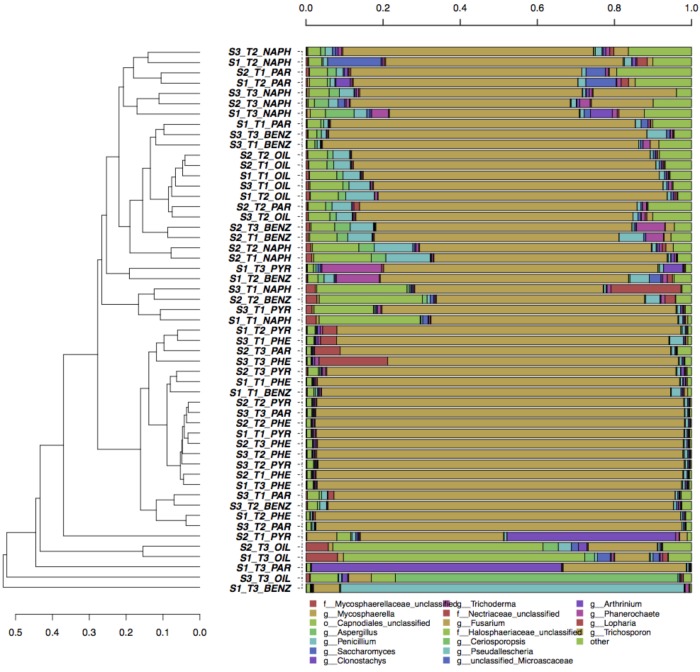
Hierarchical clustering of bacterial ITS sequences classified at the genus level. Only taxa participating with >5% in at least one sample are shown, while taxa with lower participation are grouped in the “other” sequence group.

Multivariate canonical correspondence analysis (CCA) was performed on bacterial and fungal OTUs abundance tables in order to assess how the microbial communities responded to contaminants, depth of soil collection and enrichment time (Figure 1). Among the tested factors, in the case of bacteria, pollutant was the most significant: the percentage of variance explained (36.1%, Figure 1A) was much higher than the variance explained by the soil (6.1%, Figure 1B) and by the time (8.3%, Figure 1C) factors. The bacterial communities differed among the six tested contaminants: pollutants that shared the chemical structure (e.g., the three PAHs) grouped together, while oil, benzene, and paraffin formed separate groups (Figure 1A). A similar picture was obtained by the CCA analyses performed on fungal communities: only the pollutant factor was significant (25.6% of variance explained, Figure 1D) contrary to soil and time factors. The pollutant used in the enrichment significantly affected the evolution of fungal communities: phenanthrene and pyrene enriched fungal communities formed groups that separated from those enriched from oil (Figure 1D). For both fungi and bacteria, the groups formed by soil-depth (Figures 1B–E) and time (Figures 1C–F) were mainly overlapping: this indicates that the depth, as well as the time of enrichment, did not affect the evolution of enriched microbial communities. Grouping of samples was also evaluated by UPGMA clustering of sequencing classified at genus level. Results are reported in Figure 2 for bacteria and in Figure 3 for fungi: samples are labeled according to the soil depth, followed by the pollutant and the enrichment step. Results confirmed that the relative composition in bacterial genera was partly dependent on the pollutant: several pyrene and phenanthrene samples formed two sub-clusters in a common cluster, with *Azospirillum*, *Achromobacter*, and *Pseudomonas* being the dominant genera; naphthalene enrichments were dominated by *Achromobacter* and *Pseudomonas* in similar proportions, while oil enrichments showed a dominance of *Pseudomonas* followed by *Achromobacter*. Finally, benzene and paraffin enrichments revealed a more diverse community, with high relative presences of *Acinetobacter*, *Pseudoxanthomonas*, and *Pseudomonas*. Neither the soil depth nor the time of enrichment influenced the samples clustering, confirming the CCA results presented above.

Concerning fungi, no clear clustering of genera was shown according to pollutant, soil depth, or enrichment time (Figure 3). All samples were dominated by *Fusarium* (more than 80% in average), followed by *Aspergillus*, *Penicillium*, *Trichoderma*, and *Arthrinium*.

### Comparison Between Culture-Dependent and Culture-Independent Analyses

A number of analyses were performed in order to compare the results on the diversity of bacterial and fungal strains isolated with culture-dependent and culture-independent methods. In Table 2 the relative percentages of bacterial genera retrieved from each of the three soil depths are compared. Results generally agree: *Pseudomonas* was the dominant genus in both cases, with 24.2, 25.2, and 63.5% of relative abundances in Illumina and 57.7, 48.9, and 69.6% as isolates for S1, S2, and S3, respectively. The other genera, in terms of relative percentages, were *Bacillus*, *Achromobacter*, *Sphingobacterium*, and *Pseudotrophomonas* for the culture-dependent data; *Achromobacer*, *Agrobacterium*, *Azospirilllum*, *Shinella*, *Sphingobacterium*, and *Pseudotrophomonas* for the Illumina data. It is worth noting that for some genera, the soil depth with the highest relative percentage was the same with both approaches. For instance, *Pseudomonas* reached 60% of abundance in soil three from both the approaches. There are a number of discrepancies: *Agrobacterium* sequences were above 9% in all soils while no strains belonging to this genus was isolated; and 2.2% of isolates (i.e., two strains) from S2 belong to *Helicobacter* while no sequences affiliated to this genus were detected with Illumina.

**Table 2:** Relative abundances of bacterial genera determined by Illumina sequencing of 16S amplicns or by isolation on selective media; the comparison was performed at the end of the enrichment (step III).

Bacterial genus	16S Illumina			Bacterial isolates		
	S1	S2	S3	S1	S2	S3
*Acholeplasma*	nd	nd	nd	nd	nd	4,3
*Achromobacter*	26.68	29.37	8.39	7.7	4.4	4.3
*Acinetobacter*	0.38	0.18	1.60	nd	nd	4.3
*Agrobacterium*	9.08	11.79	11.62	nd	nd	nd
*Ancylobacter*	0.30	2.53	0.40	nd	nd	nd
*Azospirillum*	4.17	2.50	0.24	nd	nd	nd
*Bacillus*	0.11	0.09	0.09	7.7	8.9	nd
*Cellulosimicrobium*	0.03	0.02	0.08	nd	nd	4.3
*Cupriavidus*	4.64	0.46	0.08	nd	2.2	nd
*Gordonia*	3.00	0.04	0.06	3.8	nd	nd
*Helicobacter*	nd	nd	nd	nd	2.2	nd
*Klebsiella*	0.08	2.63	0.08	nd	nd	nd
*Ochrobactrum*	2.89	0.67	0.32	3.8	nd	nd
*Olivibacter*	1.13	6.57	1.28	nd	nd	nd
*Paenibacillus*	0.02	0.24	0.05	nd	2.2	nd
*Pseudomonas*	24.21	25.25	63.49	57.7	48.9	69.6
*Pseudoxanthomonas*	2.95	2.62	0.66	8.7	4.4	nd
*Rhizobium*	nd	nd	nd	nd	nd	4.3
*Serratia*	0.00	0.24	0.01	nd	6.7	nd
*Shinella*	2.79	0.25	0.03	nd	nd	nd
*Sphingobacterium*	0.95	0.94	1.60	3.8	17.8	nd
*Stenotrophomonas*	1.23	0.77	1.42	7.7	2.2	8.7


*Data are expressed as percentages (among 10,065 sequences for Illumina data, among 96 strains for isolates).*

The same analyses were conducted on fungi (Table 3). Sordariomycetes was the most abundant class, with the order Hypocreales (i.e., *Acremonium*, *Clonostachys, Fusarium, Hypocrea/Trichoderma*) and Microascales (i.e., *Ceriosporopsis, Pseudoallescheria/Scedosporium*). Xylariales were instead a minor component of the detected fungi (i.e., *Arthrinium, Eutypella*). Results from culture-dependent and culture-indepentent methods confirmed each other in the case of the genus *Fusarium*, whose relative percentages were highly similar (ranging 40 and 69%). *Clonotachys*, *Pseudallescheria*, and *Trichoderma* were mostly isolated from S1 soils where also Illumina data showed the highest relative percentage. In the case of fungi too, there are some contrasting results, as fungal genera isolated in pure culture that were not detected with Illumina sequencing of ITS2 amplicons (e.g., *Bjerkandera*, *Epicoccum*, *Eutypella, Irpex, Polyporus*, and *Sulcatispora*). As for the genera *Ceriosporopsis* and *Phanerochaete*, they were detected with Illumina in significant amounts (above 5% of total sequences) but no isolates were obtained.

**Table 3:** Relative abundances of fungal genera as determined by Illumina sequencing of 16S PCR of amplicons or by isolation on selective media; the comparison was performed at the end of the enrichment (step III).

Fungal genus	ITS Illumina			Fungal isolates		
	S1	S2	S3	S1	S2	S3
*Acremonium*	0.001	0.003	nd	2.9	nd	nd
*Arthrinium*	2.0	0.4	0.3	nd	nd	nd
*Aspergillus*	1.8	2.0	0.8	8.6	8.7	19.4
*Aureobasidium*	0.9	0.3	0.3	2.9	nd	nd
*Bjerkandera*	nd	nd	nd	nd	4.3	nd
*Capnodiales_unclassified*	12.0	11.3	3.4	nd	nd	nd
*Ceriosporopsis*	0.2	0.2	14.8	nd	nd	nd
*Cladosporium*	0.2	0.6	0.1	8.6	13.0	nd
*Clonostachys*	11.0	0.6	0.5	2.9	nd	nd
*Epicoccum*	nd	nd	nd	2.9	4.3	
*Eutypella*	nd	nd	nd	nd	4.3	2.8
*Fusarium*	43.8	68.8	63.4	40.0	56.5	41.7
*Hypocrea*	nd	nd	nd	nd	nd	11.1
*Irpex*	nd	nd	nd	nd	4.3	nd
*Penicillium*	0.8	2.1	1.2	5.7	nd	2.8
*Phanerochaete*	0.5	1.7	0.3	nd	nd	nd
*Polyporus*	nd	nd	nd	nd	nd	2.8
*Pseudallescheria*	15.5	0.8	1.5	5.7	nd	nd
*Scedosporium*	0.01	0.01	0.12	nd	4.3	5.6
*Sulcatispora*	nd	nd	nd	nd	nd	2.8
*Trametes*	nd	0.1	0.04	nd	nd	2.8
*Saccharomyces*	0.9	0.7	0.1	nd	nd	nd
*Trichoderma*	3.6	0.4	0.5	20.0	nd	5.6
*Wallemia*	0.02	nd	0.02	nd	nd	2.8
*Trichosporon*	1.2	3.2	4.4	nd	nd	nd


*Data are expressed as percentages (among 10065 sequences for Illumina data, among 94 strains for isolates).*

The most abundant OTUs (i.e., those who had a relative abundance higher than 1% in at least one sample) are reported in Tables 4, 5 for bacteria and fungi, respectively. This screening criterion retained 19 OTUs for bacteria, and 21 OTUs for fungi: in both tables, the highest taxonomic affiliation is reported, together with the relative abundances in each enrichment microcosm. In the case of bacteria, most OTUs (16 out of 19) were classified at species level, two at genus and only one at the family level. Nine out of 19 OTUs were retrieved in the cultivated isolates. By comparing the data on the isolates in Table 1 with the molecular data in Table 4, it is possible to notice that the species comprising more isolates (*Pseudomonas putida*, *Achromobacter xylosoxidans*, and *Pseudomonas veronii*) were also the most abundant in terms of Illumina OTUs. As above reported for genera, also for OTUs classified at genus or species level there was a good agreement between molecular and microbiological data.

**Table 4:** Relative percentages of most abundant bacterial OTUs after the last enrichment step for each pollutant and at each of the three soil depths tested.

OTU nr	Taxonomy	Benzene			Naphtalene			Oil			Paraffin		Phenanthrene		Pyrene		
		S1	S2	S3	S1	S2	S3	S1	S2	S3	S1	S2	S1	S2	S1	S2	S3
OTU001	*Pseudomonas putida^∗^*	5.2	0.9	2.2	26.1	37.1	73.6	6.0	1.6	58.3	0.3	0.2	10.6	20.3	0.6	0.4	65.8
OTU002	*Achromobacter xylosoxidans^∗^*	7.0	2.9	4.8	46.4	20.7	4.1	10.2	20.3	6.0	8.5	11.1	23.5	23.4	26.4	51.5	6.5
OTU003	*Pseudomonas stutzeri*	0.1	0.1	0.1	0.4	0.9	0.8	1.0	0.9	5.4	0.2	0.1	0.6	1.5	0.9	0.9	1.3
OTU004	*Pseudomonas fluorescens^∗^*	0.1	0.1	0.0	1.9	4.4	3.7	16.4	20.7	9.7	0.2	0.3	1.1	3.5	0.3	0.6	2.1
OTU005	*Rhizobium petrolearium^∗^*	50.1	31.5	44.9	0.1	0.3	0.2	0.1	0.1	0.2	1.8	4.3	0.1	0.3	0.3	0.2	0.6
OTU006	*Acinetobacter calcoaceticus^∗^*	1.1	0.0	4.8	0.1	0.1	0.0	0.1	0.1	0.1	0.0	0.1	0.1	0.1	0.1	0.0	0.1
OTU008	*Azospirillum lipoferum*	0.0	0.0	0.0	0.0	0.0	0.1	0.0	0.0	0.0	0.0	0.1	0.1	10.7	4.6	0.4	0.1
OTU009	*Unclassified Comanomonadacea*	0.0	0.0	0.0	0.0	0.0	0.1	6.0	12.2	0.0	0.2	0.3	0.0	0.0	0.0	0.0	0.0
OTU010	*Ochrobactrum anthropi^∗^*	0.8	0.2	0.9	0.0	0.0	0.0	0.0	0.0	0.0	11.9	15.4	0.2	2.3	1.7	0.0	0.0
OTU011	*Rhizobium radiobacter*	0.5	4.8	1.8	0.2	0.3	0.0	0.0	0.0	0.0	0.9	3.8	1.8	1.7	1.4	19.1	0.0
OTU013	*Cupriavidus necator*	0.6	1.2	0.2	0.1	0.0	0.0	0.0	0.0	0.0	1.2	0.3	0.1	0.0	20.3	0.0	0.0
OTU014	*Gordonia alkanivorans*	0.0	0.0	0.0	0.0	0.0	0.1	0.1	0.0	0.0	16.8	2.2	0.1	0.1	0.0	0.0	0.1
OTU015	*Pseudomonas stutzeri^∗^*	0.0	0.0	0.0	0.0	0.0	0.0	24.2	0.0	0.0	0.0	0.0	0.0	0.1	0.0	0.1	0.0
OTU016	*Sphingobacterium* spp.*^∗^*	1.3	20.2	4.6	0.0	0.0	0.0	0.0	0.0	0.0	0.4	0.0	0.0	7.3	0.0	2.7	0.0
OTU017	*Bordetella* spp.	5.1	0.0	1.8	0.1	0.6	0.0	0.0	0.0	0.0	0.5	2.9	0.1	0.5	0.2	0.2	0.0
OTU018	*Pseudoxanthomonas mexicana^∗^*	2.7	1.1	1.6	0.0	1.1	0.1	0.0	0.0	0.0	0.8	0.3	2.9	2.9	5.4	2.8	0.1
OTU021	*Sinorhizobium* spp.	2.0	2.2	1.2	0.0	0.2	0.0	0.0	0.0	0.0	1.5	7.0	0.0	0.1	0.2	0.1	0.0
OTU023	*Xanthobacter flavus*	0.0	0.1	0.1	0.0	0.0	0.0	0.0	0.0	0.0	5.6	21.1	0.0	0.1	0.0	0.1	0.0
OTU024	*Pseudomonas resinovorans*	0.1	0.3	0.0	0.5	0.0	0.1	0.4	0.5	2.1	0.0	0.0	12.0	0.2	1.0	0.6	0.1
OTU025	*Azospirillum thiophilum*	0.0	0.0	0.0	0.0	0.0	0.0	0.0	0.0	0.0	0.0	0.0	17.7	0.0	0.0	0.0	0.5


*OTUs that represent more than 1% of total bacterial community in at least one sample are presented. OTUs that were also detected among isolates are highlighted with an asterisk.*

**Table 5:** Relative percentages of most abundant fungal OTUs after the last enrichment step for each pollutant and at each of the three soil depths tested. OTUs that represent more than 1% of total fungal community in at least one sample are presented.

OTU nr	Taxonomy	Benzene			Naphtalene			Oil			Paraffin			Pheanthrene			Pyrene	
		S1	S2	S3	S1	S2	S3	S1	S2	S3	S1	S2	S3	S1	S2	S3	S1	S2
OTU002	*Fusarium oxysporum^∗^*	3.5	39.1	16.6	29.4	23.4	36.3	4.7	4.6	3.3	9.8	15.3	13.4	39.3	56.4	63.5	56.4	78.3
OTU003	*Cladosporium* spp.*^∗^*	1.3	7.5	2.4	5.6	2.5	6.4	71.2	61.8	8.4	1.1	1.4	1.8	1.5	1.7	1.4	2.0	3.4
OTU004	*Fusarium* spp.^∗^	0.9	5.1	2.1	5.5	5.8	6.5	1.3	3.5	0.9	9.8	64.9	30.2	35.9	26.1	2.0	2.9	3.4
OTU001	*Fusarium solani^∗^*	2.6	21.8	64.3	13.2	22.6	15.1	2.4	10.1	1.8	12.7	4.4	51.2	18.5	11.7	7.6	7.9	7.6
OTU007	*Unidentified Halosphaeriacea*	0.2	0.0	0.0	0.2	0.2	0.2	0.2	0.2	80.4	0.1	0.2	0.1	0.2	0.3	0.2	0.2	0.2
OTU014	*Fusarium merismoides*	0.0	0.0	0.0	0.2	0.1	0.4	0.1	0.1	0.0	0.1	0.1	0.2	0.2	0.2	20.2	0.4	0.1
OTU005	*Pseudallescheria* spp.	89.0	0.7	6.6	1.1	1.5	0.6	0.6	0.5	0.3	0.7	1.0	0.9	0.7	0.6	0.8	1.0	0.9
OTU008	*Apiospora montagnei*	0.2	0.1	0.1	5.9	0.7	0.6	0.3	0.4	0.1	0.2	0.3	0.4	0.5	0.3	0.4	5.4	0.8
OTU012	*Aspergillus flavus*	0.1	2.8	0.2	5.4	3.2	1.6	0.1	0.2	0.0	0.0	0.1	0.1	0.1	0.1	0.1	0.6	0.2
OTU030	*Saccharomyces cerevisiae*	0.1	0.2	0.1	0.7	1.8	0.2	3.6	1.8	0.2	0.1	0.1	0.0	0.1	0.1	0.1	0.6	0.1
OTU009	*Penicillium* spp.*^∗^*	0.1	4.6	0.8	1.3	1.5	2.7	0.2	1.9	0.2	0.1	0.1	0.3	0.1	0.1	0.1	0.3	0.2
OTU010	*Trichoderma harzianum^∗^*	0.2	0.2	0.1	5.1	0.7	0.6	0.2	0.3	0.1	0.3	0.3	0.3	0.5	0.3	0.8	16.0	0.7
OTU006	*Fusarium cuneirostrum*	0.1	0.1	0.1	0.5	0.3	0.3	0.3	1.9	1.4	64.0	0.3	0.2	0.2	0.5	0.4	0.4	0.4
OTU028	*Aureobasidium pullulans^∗^*	0.0	0.5	0.6	4.0	1.2	0.4	0.0	0.1	0.1	0.0	0.0	0.0	0.0	0.1	0.0	0.5	0.3
OTU024	*Trichosporon* spp.	0.1	0.7	0.0	3.1	3.4	4.5	0.0	0.0	0.0	0.0	0.1	0.0	0.1	0.1	0.0	0.1	0.1
OTU019	*Galactomyces geotrichum*	0.0	0.1	0.2	0.5	3.3	0.1	0.0	0.0	0.0	0.0	0.0	0.0	0.0	0.0	0.0	0.0	0.0
OTU021	*Cadophora malorum*	0.0	0.1	0.3	1.3	0.1	0.3	3.5	1.0	0.1	0.1	1.8	0.0	0.1	0.1	0.1	0.1	0.0
OTU011	*Trichosporon loubieri*	0.0	1.8	0.1	3.0	10.3	16.2	0.1	0.1	0.1	0.1	0.2	0.0	0.1	0.1	0.1	0.3	0.2
OTU018	*Phanerochaete chrysosporium*	1.0	7.3	0.5	1.5	2.7	0.8	0.7	0.0	0.5	0.0	0.0	0.0	0.1	0.1	0.1	0.2	0.2
OTU114	*Fusarium solani^∗^*	0.0	0.1	0.1	0.2	3.9	0.0	0.0	0.0	0.0	0.0	0.0	0.0	0.0	0.0	0.0	0.0	0.0
OTU132	*Fusarium* spp.*^∗^*	0.1	0.1	0.0	0.2	0.1	0.1	0.0	0.0	0.1	0.1	1.0	0.0	0.2	0.2	1.2	3.1	0.1
OTU033	*Hypocrea gamsii*	0.0	0.0	0.0	0.1	0.1	0.0	2.5	0.0	0.1	0.0	0.1	0.0	0.1	0.0	0.0	0.0	0.0


*OTUs that were also detected among isolates are highlighted with an asterisk.*

Regarding fungi, the rate of OTUs that could be classified at species level is similar to the one for bacteria: 14 out of 21. The number of OTUs also detected among isolates was instead slightly lower, 9 isolates (Table 5). It is worth noting that, as for bacteria, the taxa comprising more isolates were also the most abundant OTUs: that was the case for *Fusarium oxysporum*, *Cladosporium* spp., and *Fusarium solani*. These data highlighted the different adaptation abilities developed by fungi: while species of the genus *Fusarium* were abundant and widely distributed among pollutants and depth, other fungi seemed to be particularly adapted to individual contaminant. For instance, the relative percentage of *Cladosporium* spp. and *Pseudoallescheria* spp. was significantly high only in the presence of oil and benzene, respectively.

## Discussion

Since the discovery of the great plate count anomaly in the middle ‘80s of the last century (Staley and Konopka, 1985), microbiologists are facing the issue of non-cultivable microorganisms and the underestimation of the actual microbial community by means of direct isolation techniques. This has become more and more relevant with the advent of HTS techniques, and it is of utmost importance in the environmental microbiology sector of bioremediation, where studies must rely on the isolation and identification of bacterial and fungal strains to be inoculated in soil for efficient degradation of target pollutants (Megharaj et al., 2011). These strains are usually obtained through enrichment procedures, aimed at isolating autochthonous microorganisms capable of using the pollutants as sole carbon sources (Thompson et al., 2005). Although many data are available in literature about the assessment of microbial community dynamics following the addition of enriched microbial strains (MacNaughton et al., 1999; Aburto-Medina et al., 2012; Fuentes et al., 2014), little is known on the developments that occur within the bacterial and fungal communities during the enrichment steps. Furthermore, to the best of our knowledge, no attempt was made to assess how soil samples with a high petroleum hydrocarbons content and collected at different depths affect the organization of the bacterial and fungal communities.

In this work, we assessed whether the pollutant and the collection depth had significant effects on the microbial community arrangements. Enrichments were performed using five pollutants (three PAHs, benzene, and paraffin) and a complex mixture (i.e., crude oil collected from the polluted site). The results obtained (Figure 1) highlighted that for both bacteria and fungi, the pollutant partially shaped the enriched communities. Although it was difficult to highlight separate clusters according to the pollutants, some differences were observed as bacteria grown in the presence of paraffin and benzene or with PAHs, and fungi grown with paraffin or PAHs (Figure 1). This is not surprising, if we consider that different metabolic pathways are involved in the degradation of aliphatic or aromatic hydrocarbons, but that single strains can be also equipped with both pathways (Whyte et al., 1997). The microbial community responded uniquely to the presence of crude oil: only those strains, highly adapted to this extreme and toxic environment, were capable of colonizing this ecological niche.

According to literature, a relation between pollutants load and microbial communities can be drawn. Several studies assessed a different distribution of PAHs and other oil pollutants along soil depths (Cousins et al., 1999; Ping et al., 2007). It is well-known that microbial community organization is affected by soil depths, commonly causing a decline in biomass and diversity from the surface downward (Blume et al., 2002; Fierer et al., 2003; Oehl et al., 2005). As far as we know, no experiments were previously conducted to assess the impact of the soil samples depths on the community structure and enrichment outcomes. Contrary to this general background, in the present study, neither the soil depth nor the enrichment step had significant effects on the community structure (Figures 1–3). Regarding fungi, this could be explained by their physiological growing capabilities. Thanks to their hyphae growth, they can widely colonize the soil, creating a homogenous mycoflora in the space (Hesham et al., 2017).

The total load of bacteria and fungi was lower than unpolluted soils (Supplementary Table S1), although no differences were observed among the collection depths: a strong and long-lasting pollution of this site may shape a microbial community adapted to the pollutants pressure mostly composed by oil-utilizing strains. As illustrated in Figures 2, 3, differences among samples are more evident for bacteria. However, it is not possible to define clear clusters based on soil depths; the structure of the microbial community was not dependent by the collection point. Since the microbial community is selected by the total content of the pollutants, we can speculate that, in this soil, a pollutants threshold has been reached in the surface topsoil: beyond this level, only the selected and adapted microflora can survive. Indeed, in a less polluted site, the presence of both aerobic and anaerobic microorganisms decreased with the vertical soil profile (Biró et al., 2014). Regarding the enrichment steps, it was surprising to find that already after 1 week of enrichment, the communities were similar to those found after 4 weeks. This indicates that when dealing with strong polluted soils, as in this study, a single enrichment step may be enough.

A total of 95 bacterial and 94 fungal strains were identified. These strains were well-distributed along soil depths and pollutant (Supplementary Tables S2, S3). The taxonomical identification of bacteria confirmed the selection of specific bacterial communities following the addition of different hydrocarbons, indicating a high specialization of bacterial taxa involved in their degradation. *Proteobacteria, Firmicutes*, and *Sphingobacteria* were the prevailing bacterial phyla: all these phyla comprised several phylogenetic groups involved in the aerobic degradation of hydrocarbons (Vinas et al., 2005; Head et al., 2006; Yang et al., 2012; Yergeau et al., 2012). *Proteobacteria* was also found as the most abundant phylum distributed in an environment contaminated with petroleum muck and in activated biomass from a petrochemical industry wastewater sample (Joshi et al., 2014; Yadav et al., 2015). This phylum is divided into five major classes, all with oil-degrading genera (Gao et al., 2014). On the whole, isolated bacteria where ascribed mainly to genera belonging to Gamma-proteobacteria class, whose dominance within the enriched microcosms can be expected since it comprises many of the hydrocarbonoclastic bacteria responsible for the first steps of hydrocarbons degradation (Head et al., 2006; Yakimov et al., 2007; Barbato et al., 2016).

Bacterial species of the genera *Sphingobacterium, Achromobacter, Pseudomonas, Stenotrophomonas*, and *Bacillus*, isolated from this crude-oil contaminated soil, showed close lineage with previously reported hydrocarbons degrading members of same genera and most of them have been reported earlier as hydrocarbon degraders (Widdel and Rabus, 2001; Roy et al., 2002; Foght, 2008; Chandra et al., 2013; Varjani et al., 2015; Varjani and Upasani, 2016). Members of the genus *Pseudomonas* are widespread environmental microorganisms isolated from a variety of natural sources (Velmurugan et al., 2011) and well-known biodegraders, capable of metabolizing a range of compounds (Kostka et al., 2011; Mulet et al., 2011; Mahjoubi et al., 2013).

According to our results, the PAHs-degrading isolates in soil belong to the *Sphingomonas* and *Pseudomonas* bacteria. *Pseudomonas* usually contain *nah*-like genes that encode for the dioxygenase subfamily; the products of these genes are capable of degrading low-molecular- weight PAHs (Johnsen et al., 2007) and producing a rhamnolipid biosurfactant in soils contaminated with petroleum hydrocarbons (Sadouk et al., 2008; Xia et al., 2014). Unlike other gram-negative bacteria strains, members of the genus *Sphingomonas* are able to degrade a wide range of natural and xenobiotic compounds (Eguchi et al., 1996; Pinhassi and Hagstrom, 2000). Three strains of *Serratia marcescens* were also isolated from the enriched microcosms with the crude oil of the contaminated site, supporting previous evidences of degradation of crude oil and petroleum products by species of *Serratia* (De La Fuente et al., 1991; Rojas-Avelizapa et al., 2002; Wongsa et al., 2004; Rajasekar et al., 2007).

Generally, fungi involved in the degradation of PAHs include ligninolytic and non-ligninolytic fungi. Most of the strains isolated after the enrichment steps belonged to the phylum Ascomycota, although few Basidiomycota were isolated. The dominance of Ascomycota in polluted soil has been extensively acknowledged: Zhang et al. (2018) reported that Ascomycota represented up to 73–96% of the total 18S sequences of a coking area soil; eight of the 10 strains isolated from a PAHs contaminated pond were Ascomycetes and were also able to remove anthracene (Aranda et al., 2017). Finally, an oilfield with a history of 50 years pollution influenced mainly the bacterial community, whereas fungi, mostly related to this phylum, were abundant, demonstrating to be less sensitive to soil PAHs (Zhou et al., 2017).

Basidiomycota represented only the 5.3% of the total isolates. Similarly, in a toxic coking area, only a small fraction of the isolates belonged to Basidiomycota (0.34–7.0%) (Zhang et al., 2018). Besides *Bjerkandera adusta, Irpex lacteus, Wallemia mellicola, Polyporus gayanus, Trametes gibbosa*, the presence of species of *Lopharia, Phanerochaete*, and *Trichosporon* was assessed by HTS. Among them, *T. gibbosa, P. gayanus*, and *W. mellicola* were isolated for the first time in hydrocarbons polluted soil.

On the other hand, Mucoromycota were absent in this contaminated soil. Unlike Morais et al. (2016) who found more than 50% of Mucoromycota in a petroleum contaminated costal site, we did not identified any organism belonging to this phylum. Mucoromycota represented a small fraction of the isolates also in an estuarine sediment contaminated mainly by PAHs (Da Silva et al., 2003) and in heavy crude oil-contaminated soil (Zafra et al., 2014). In the present study, Mucoromycota could be included in the “unclassified” group (Figure 3), even though they would be a negligible fraction of the mycoflora.

All samples were dominated by *Fusarium*, followed by *Aspergillus*, *Penicillium*, and *Trichoderma*. The identification of these genera as polluted soil inhabitants has been already discussed elsewhere. Sordariomycetes is the most represented class with *Acremonium, Arthrinium, Clonostachys, Eutypella, Fusarium, Hypocrea/Trichoderma*, and *Pseudoallescheria/Scedosporium*. Fungal taxonomy analysis of an oil-contaminated soil detected almost 25% of Sordariomyecetes (Morais et al., 2016). *Trichoderma harzianum* (Da Silva et al., 2003), *Scedosporium apiospermum* and *Acremonium* sp. (Chaıneau et al., 1999; Zafra et al., 2014), *Fusarium oxysporum* (Marchand et al., 2017), and *Fusarium solani* (Chaıneau et al., 1999) were also isolated from contaminated soil or sediments. *Fusarium* (three strains), *Trichoderma* (three strains), and *Pseudallescheria* (two strains) represented the 40% of the isolates from heavy and extra-heavy crude oil polluted soils (Naranjo et al., 2007). Zhou et al. (2017) detected more than 20% of *Gibberella* species in an oilfield site. Since *Gibberella* is the teleomorph of the majority of *Fusarium* species (Leslie and Summerell, 2008) we can state that this finding is in agreement with our observations. The isolation of 42 strains of *Fusarium*, among which 64% were ascribable to *F. solani*, opens to intriguing solutions for their application in bioremediation processes. Indeed *F. solani* isolated from oil polluted soil by enrichment method using phenanthrene as the sole source of carbon was capable of degrading a mixture of low and high molecular weight PAHs (up to 84.8%) (Hesham et al., 2017). To the best of our knowledge, this is the first time that a strain of *Eutypella scoparia* has been isolated from a polluted soil. Previous researches reported the isolation of this species only in a non-polluted Arctic soil (Bergero et al., 1999; Liu et al., 2014).

Surprisingly, Morais et al. (2016) did not found Eurotiomycetes in a contaminated soil. On the contrary, in the present study, the fungal community was rich of *Aspergillus* and *Penicillium. Aspergillus* and *Penicillium* strains have been already isolated in several polluted samples (Chaıneau et al., 1999; Da Silva et al., 2003; Zafra et al., 2014; Al-Hawash et al., 2018). Data on soil isolation of *Aspergillus niger* (Chaıneau et al., 1999)*, Aspergillus fumigatus* (Chaıneau et al., 1999; Zafra et al., 2014), *Aspergillus terreus* (Da Silva et al., 2003), *Aspergillus flavus, Aspergillus niger, Aspergillus nomius* (Zafra et al., 2014), *Aspergillus sydowii* (Tian et al., 2016), *Aspergillus versicolor* (Šimonovièová et al., 2017) have been reported although few information deals with the here-detected species of *Aspergillus* (i.e., *A. creber, A. jensenii, A. protuberus*). For instance, *A. creber* and *A. jensenii*, described as new species in Jurjevic et al. (2012), are mostly known as air-born contaminants or of clinical relevance (Micheluz et al., 2016; Siqueira et al., 2016); *A. protuberus* has been isolated from air and the mussel *Mytilus edulis galloprovincialis* (Siqueira et al., 2016; Klas et al., 2018). There is no evidence in literature of these species as a terrestrial inhabitant. Although already detected in soil, *Aspergillus sclerotiorum* and *Aspergillus waskmanii* have been only associated to not-contaminated lands (Nesci et al., 2006; Phainuphong et al., 2017) or as part of the rhizosphere of tussock (Di Menna et al., 2007). Although this is the first time a *Penicillium catenatum* is found in a polluted soil, it must be considered that the identification of soil fungi is not always accurate at species level (Al-Hawash et al., 2018).

A thorough comparison was performed between the relative abundances of bacterial and fungal strains. The comparisons carried out at genus level (Table 2 for bacteria, Table 3 for fungi) and at OTU level (Table 4 for bacteria, Table 5 for fungi) showed an agreement between the culture-dependent and the culture-independent approaches. In most cases, species level was reached, thus confirming that the Illumina sequencing of the two hypervariable V3–V4 regions and of the partial ITS2 are sufficient to reach the species level for bacteria and fungi respectively. The species with more isolates (such as *P. putida*, *A. xylosoxidans* and *Ochromobactrum anthropi* for bacteria, *F. oxysporum* and *F. solani* for fungi) were also the dominant OTUs, thus showing that the culturing methods reflect the relative composition of total communities. Furthermore, the two approaches also agree for the less abundant genera: this is expected since enrichment procedures indeed select for the cultivable species among the total bacterial and fungal communities that is originally present in the polluted soil. A novel aspect shown in our work is that the two approaches had detection levels that were generally in good agreement. Composition of the actual microbial community and are not much subjected to biases.

## Conclusion

In the present study, we evaluated the evolution of bacterial and fungal communities enriched from polluted soil by culture-independent and dependent methods. The results showed that already after 1 week of enrichment, both bacterial and fungal communities resembled those found after 4 weeks, and that the soil depth did not influence the evolution of microbial communities, contrary to the pollutant used. Isolation results were in agreement with HTS molecular data, indicating that the strains isolated reflected the microbial composition of the enriched consortia. Future studies will focus on the biodegradation abilities of the isolates microorganisms.

## Author Contributions

EP and GV ideated and supervised the study. GS, FS, and AP performed the experiments and wrote the paper. A-LB and TR performed the experiments. CG carried out chemical analyses.

## Conflict of Interest Statement

The authors declare that the research was conducted in the absence of any commercial or financial relationships that could be construed as a potential conflict of interest.

## Footnotes

1 http://hannonlab.cshl.edu/fastx_toolkit/

2 http://www.R-project.org
