# Supplementary material for: Molecular and Microbiological Insights on the Enrichment Procedures for the Isolation of Petroleum Degrading Bacteria and Fungi
Source: Front Microbiol. 2018 Oct 30;9:2543. doi: 10.3389/fmicb.2018.02543 (PMC6218658; doi:10.3389/fmicb.2018.02543)
Supplement: Supplementary file 1 [file Table_1.DOCX]

**Table S1.** Chemical concentrations (mg Kg^-1^) of pollutants and total bacterial and fungal counts in the three soils depth samples used as inocula for the enrichments.

|  | **S1 (0-1m)** | **S2 (1-2m)** | **S3 (2-3m)** |
| --- | --- | --- | --- |
| 2-metilnaphthalene | 3.30 | 9.03 | 102 |
| 1-metilnaphthalene | 5.81 | 7.98 | 47.50 |
| naphthalene | 6.42 | 8.16 | 105 |
| acenaphtilene | 0.18 | 0.38 | 0.32 |
| acenaphthene | 9.64 | 6.43 | 15.50 |
| fluorene | 18.10 | 8.55 | 19.20 |
| phenanthrene | 96.80 | 125 | 103 |
| anthracene | 7.81 | 6.48 | 9.12 |
| fluoranthene | 54.90 | 66.50 | 59.00 |
| pyrene | 39.00 | 45.20 | 40.80 |
| benzo(a)anthracene | 10.70 | 12.60 | 11.60 |
| chrysene | 6.63 | 7.63 | 6.53 |
| benzo(b+j)fluoranthene | 4.84 | 6.54 | 4.85 |
| benzo(k)fluoranthene | 1.81 | 2.22 | 1.75 |
| benzo(a)pyrene | 2.43 | 3.12 | 2.49 |
| indeno | 2.49 | 2.32 | 1.38 |
| dibenzo(ac+ah)anthracene | 0.34 | 0.54 | 0.34 |
| benzo(ghi)perylene | 1.15 | 1.87 | 1.08 |
| dibenzo(al)pyrene | 0.45 | 1.08 | 0.41 |
| dibenzo(ae)pyrene | 0.14 | 0.27 | 0.15 |
| dibenzo(ai)pyrene | 0.04 | 0.14 | 0.06 |
| dibenzo(ah)pyrene | 0.02 | 0.06 | < 0.01 |
| total PAHs | 273 | 322 | 532 |
| benzene | 23.51 | 39.12 | 16.40 |
| toluene | 10.50 | 18.17 | 16.60 |
| ethylbenzene | 0.35 | 0.85 | 1.30 |
| xylenes | 6.52 | 12.34 | 9.46 |
| styrene | 0.61 | 0.10 | 0.03 |
| hydrocarbons C≤12 | 6.39 | 11.76 | 37.48 |
| hydrocarbons C>12 | 774 | 420 | 455 |
| residues at 105°C % | 83.61 | 83.23 | 83.65 |
| Total bacterial counts at 37°C | 3.7*10^7^ cfu g^-1^ | 1.1*10^8^ cfu g^-1^ | 4.1*10^7^ cfu g^-1^ |
| Total fungal counts at 37 °C | 1.2*10^4^ cfu g^-1^ | 1.7*10^4^ cfu g^-1^ | 1.3*10^2^ cfu g^-1^ |

**Table S2.** Identification of the bacteria isolated from each depth of contaminated soil. The number of isolates per genera/species is indicated in parenthesis.

| **Soil S1 (0-1m)** | **Soil S2 (1-2m)** | **Soil S3 (2-3m)** |
| --- | --- | --- |
| *Achromobacter sp. (1)* | *Achromobacter xylosoxidans (2)* | *Achromobacter sp. (1)* |
| *Bacillus subtilis (2)* | *Bacillus subtilis (1)* | *Pseudomonas sp. (10)* |
| *Ochrobactrum anthropi (1)* | *Bacillus xiamenentis (1)* | *Acholeplasma vituli (1)* |
| *Pseudomonas fluorescens (4)* | *benzo[a]pyrene-degrading bacterium (1)* | *Acinetobacter calcoaceticus (1)* |
| *Pseudomonas mosselii (1)* | *Cupriavidus campinensis(1)* | *Cellulosimicrobium sp. (1)* |
| *Pseudomonas putida (4)* | *Helicobacter sp. (1)* | *Pseudomonas fluorescens (5)* |
| *Pseudomonas sp. (8)* | *Paenibacillus sp. (1)* | *Pseudomonas putida (6)* |
| *Pseudoxanthomonas mexicana (2)* | *Pseudomonas aeruginosa (2)* | *Pseudomonas veronii (1)* |
| *Sphingobacterium multivorum (1)* | *Pseudomonas fluorescens (4)* | *Rhizobium petrolearium (1)* |
| *Stenotrophomonas acidaminiphila(1)* | *Pseudomonas mosselii (1)* | *Stenotrophomonas acidaminiphila (1)* |
| *Stenotrophomonas maltophilia (1)* | *Pseudomonas putida (4)* | *Stenotrophomonas maltophila (1)* |
|  | *Pseudomonas sp. (10)* |  |
|  | *Pseudomonas veronii (2)* |  |
|  | *Pseudoxanthomonas indica (2)* |  |
|  | *Serratia marcescens (3)* |  |
|  | *Sphingobacterium multivorum (9)* |  |
|  | *Stenotrophomonas acidaminiphila (1)* |  |

**Table S3.** Identification of the fungal isolated from each depth of contaminated soil. The number of isolates per species is indicated in parenthesis.

| **Soil S1 (0-1m)** | **Soil S2 (1-2m)** | **Soil S3 (2-3m)** |
| --- | --- | --- |
| *Acremonium sclerotigenum (1)* | *Aspergillus versicolor (2)* | *Aspergillus jensenii (1)* |
| *Aspergillus creber (1)* | *Bjerkabndera adusta (1)* | *Aspergillus protuberus (1)* |
| *Aspergillus sclerotiorum (1)* | *Cladosporium cladosporioides (1)* | *Aspergillus versicolor (4)* |
| *Aspergillus sydowii (1)* | *Cladosporium perangustum (2)* | *Aspergillus waksmanii (1)* |
| *Aureobasidium pullulans (1)* | *Epicoccum nigrum (1)* | *Eutypella scoparia (1)* |
| *Cladosporium cladosporioides (3)* | *Eutypella scoparia (1)* | *Fusarium oxysporum (4)* |
| *Clonostachys rosea (1)* | *Fusarium oxysporum (7)* | *Fusarium solani (8)* |
| *Epicoccum nigrum (1)* | *Fusarium solani (4)* | *Fusarium solani/keratoplasticum (3)* |
| *Fusarium oxysporum (4)* | *Fusarium solani/keratoplasticum (2)* | *Hypocrea lixii (4)* |
| *Fusarium solani (10)* | *Irpex lacteus (1)* | *Penicillium crustosum (1)* |
| *Penicillium brevicompactum (1)* | *Scedosporium apiospermum (1)* | *Polyporus gayanus (1)* |
| *Penicillium catenatum (1)* |  | *Scedosporium apiospermum (1)* |
| *Pseudoallescheria boydii (2)* |  | *Scedosporium dehoogii (1)* |
| *Trichoderma harzianum (7)* |  | *Sulcatispora acerina (1)* |
|  |  | *Trametes gibbosa (1)* |
|  |  | *Trichoderma harzianum (2)* |
|  |  | *Wallemia mellicola (1)* |
